# Dropsy; Its Therapeutics

**Published:** 1904-06

**Authors:** Frank F. Hoffmann

**Affiliations:** Chicago, Ill., Surgeon to American Hospital and Frances E. Willard National Temperance Hospital


### Dropsy; Its Therapeutics.

By FRANK F. HOFFMANN, M. D., Chicago, Ill.,
Surgeon to American Hospital and Frances E. Willard National Temperance Hospital.

DROPSY is a collection of fluid in any of the cavities or the cellular tissue of the body, and may be conveniently classified as edema, anasarca, ascites, hydrothorax, and hydropericardium. Although usually an acquired condition it may be congenital. In this discussion, however, we will refer only to acquired dropsy due to cardiac, renal, hepatic, or peritoneal disease. The dropsy of cardiac disease may be of acute or chronic origin. Effusion into the pericardium may result from an acute pericarditis, usually of rheumatic character, or it may occur in the course of chronic cardiac or renal disease. Dropsy due to valvular lesions of the heart usually occurs at a late period when compensatory disturbances have set in, and is generally complicated with renal disease.

In hydrothorax, due to acute pleurisy, the fluid is at first of a thin serous character, and later becomes serofibrinous. Aside from those cases in which the pleurisy is due to exposure to cold or complicates pneumonia, it is frequently of tuberculous character. The latter variety is apt to develop insidiously, and the exudate is liable to assume a purulent nature. Acute infectious fevers may also be accompanied by hydrothorax. Chronic hydrothorax may be a sequence of an acute pleurisy, or it may be of tubercular origin, or a late complication of diseases of the heart or kidneys. Ascites or hydroperitoneum may be due to peritonitis, Bright's disease, cirrhosis of the liver, or valvular lesions of the heart. It is apt to be especially marked in hepatic or renal disease.

General dropsy, or anasarca, is not apt to occur in cirrhosis of the liver, but it is very common in renal affections. In cases of valvular lesions of the heart we may get a general edema independent of disease of the kidneys, although chronic nephritis is present in 70 per cent. of the cases. I recently observed a case of cyst of the gallbladder in which adhesions had taken place and in which the diagnosis from ascites was somewhat difficult. The nature of the case, however, was established by an exploratory aspiration, which revealed the presence of bile. Chylous ascites is a peculiar condition not due to obstruction of the portal circulation, but to a rupture of the thoracic duct, caused by ulceration, cancer, or the like. Chylous fluid may also enter the pleural cavity, usually on the left side.

With regard to the treatment of dropsy it must be remembered that it is not a disease but a symptom, although it often assumes an importance sufficient to make special treatment necessary. We must remove the cause if possible. In acute inflammatory affections of the heart and pericardium, I have often been able to witness a control of the inflammation in from 6 to 15 hours upon giving the tincture of bryonia, one drop every two hours, or three drops of the fluid extract; and in such the condition did not become chronic. As this article is limited to the discussion of dropsy, I shall not, however, enter into the treatment of those diseases of the heart, liver and kidneys of which it is a manifestation.

Where the effusion is localised, as in hydropericardium, it may be necessary to aspirate, which is best performed in the fourth interspace, about two inches to the left of the sternum, always after the most thorough antiseptic preparation. In effusions into the pleural sac thoracentesis may be required if medicinal treatment fails, and this is to be followed by strapping the chest.

The indications for abdominal paracentesis are large effusions with dyspnea, pain and cardiac disturbances. It should be performed a little to one side of the median line and about three inches above the pubes, if necessary, first aseptically catheterising the bladder. Aside from the mechanical removal of dropsical effusions we may resort to elimination by way of the bowels, the skin, and the kidneys, or to a combination of these measures. Elimination by way of the bowels may be accomplished by the administration of salines, such as epsom or rochelle salts, in doses of two drams every four hours, or with compound jalap powder or calomel. Other drugs for this purpose are elaterin, 1-10 grain, or extract of elaterium, 1-6 grain, once or twice daily, or croton oil in one drop doses pro die. These powerful evacuants should be reserved for cases in which the dropsy requires urgent removal, as in renal affections. Elimination through the skin is best promoted by hot tub baths or vapor baths, assisted by hot drinks.

In most instances, however, the use of diuretics is necessary, and we must distinguish between those which act upon the heart and arterial system, and those which act directly upon the kidneys. The selection of one or the other class will depend, of course, upon the cause of the dropsy. If it is of cardiac origin, the remedies selected should be those which are known to regulate the action of the heart and to increase the arterial tonicity and thus improve the circulation. On the other hand, if the dropsy is due to advanced renal disease it is important to exclude from the treatment those drugs which act directly upon the kidneys, in order to avoid any increased irritation of organs already functionating badly. Among the cardiac diuretics, so-called, digitalis is the most prominent. The infusion is commonly employed in doses of one-half to one ounce, and the best results are obtained in cases in which the pulse is rapid, weak, and irregular. After its effect has manifested itself it may be continued in half ounce doses, *t. i. d.*, for some time, always discontinuing its use if nausea appears.

Strophanthus may also be carefully employed in doses of one to four minims of the tincture, until the heart's action becomes regular. Its use should be suspended if headache and a sense of constriction manifest themselves. Convalleria is also recommended by some authors, its action upon the heart resembling that of digitalis. Caffein enjoys some popularity as a diuretic, and acts both upon the heart and kidneys. It may be given in doses of one to three grains, every four hours, and continued until free diuresis is produced, when it generally loses its effect. Squills is an oldtime diuretic which sometimes acts satisfactorily when digitalis fails. Its effect is exerted both upon the heart and kidneys, and in cases in which the latter are diseased it must be used very cautiously.

Among the diuretics which act directly upon the kidneys we have the alkaline vegetable salts, such as the bitartrate, acetate, or citrate of potassium. These seem to promote elimination by stimulating the renal circulation and increasing osmosis. If employed in moderate doses they are usually well tolerated, and are often combined to advantage with the cardiac group of diuretics. Other renal diuretics are polytricum, in 30-drop doses of the tincture, which I have found of value in the dropsies of cardiac origin; juniper, turpentine, and copaiba, the last three, however, being rarely employed owing to their irritating effect. The best type of renal diuretics is represented by theobromine and its derivatives. Their action seems to be exerted directly upon the renal secreting structures, without any influence upon the heart or general circulation, and for these reasons they have been largely employed in dropsy of cardiac affections and of those chronic renal troubles in which the function of the kidneys is not too greatly injured.

Theobromine itself is quite irritating and is liable to cause gastric disturbances, and on account of this theobromine sodium salicylate has largely taken its place. As the salicylic acid in its composition, however, is not free from irritating effects upon the kidneys and stomach, a still more eligible method of administering theobromine is theobromine sodium acetate, or agurin. In this drug the influence of theobromine is supplemented by that of the acetate of sodium, which, as already said, is in itself an excellent diuretic. The following case will serve to illustrate the action of agurin.

J. S., 38 years old, suffered from organic heart disease, with edema and ascites three years ago. The stomach was very irritable. Convalleria was first given in 3-drop doses of the fluid extract, and this acted very promptly, increasing the elimination of urine to four times the amount within 24 hours. The dropsy returned at the end of six months, when a trial was made of agurin in 2-grain doses, three times daily. Its action was even more pronounced than that of convalleria, and in 24 hours the urine had increased from 600 c.cm. to 5,000 c.cm. This was followed by a slight decline, after which the amount again increased to the former level, remaining at 3,000 c.cm. for two weeks. The bowels were kept open with magnesium sulphate and podophyllin, these producing six to twelve watery stools daily. Under this treatment the dropsy completely subsided, and has failed to return.

In three other cases I observed the same marked results, excepting one with double cardiac aneurism and enormous hypertrophy of the heart, with irregular pulse. Here strophanthus, one-quarter drop of the specific tincture, was administered, after which agurin promptly increased the flow of urine.

In atrophic cirrhosis of the liver with ascites the effect of the agurin is not so rapidly developed nor so persistent, but under the administration of five grains, four times daily, for three days, in every case there was a gradual increase of the urine up to 5,000 c.cm., after which it dropped to 3,000 c.cm., and was kept at this level for some time by five grains given only at night. Abdominal paracentesis was also performed on a number of occasions, the amount of fluid removed varying from 1 to 4 gallons. One case left the hospital in six months able to resume his work, having been confined to his bed for two months during that period. The other patient left after three weeks' treatment, somewhat improved, and passed from observation. In hypertrophic cirrhosis of the liver with jaundice and with some effusion into the abdomen, agurin was equally efficacious. It seemed to have a beneficial effect also in passive congestion of the liver, probably by favoring elimination of toxins from the body.

In dropsical effusions in the pericardium and pleural cavity agurin is a useful diuretic given for some time in small doses. In a case of left-sided pleural effusion in which aspiration was performed, with removal of the serous fluid, the patient refused medication and the cavity rapidly filled again. In a second aspiration a serous fluid was removed, and the patient was now prevailed upon to take a tonic of cocoa wine, and was given agurin in 5-grain doses, four times daily. The urine almost immediately increased to twice its volume, and the diuresis was maintained for some time, a solid diet being given for a week. At the end of five days agurin was stopped. At that time the temperature and pulse were nearly normal, although respiration was still somewhat limited. The urinary quantity remained above the normal for several days after discontinuing the drug. The patient is now in perfect health.

In acute nephritis agurin is of no benefit, and its diuretic action cannot be depended upon. In a case under my observation of general anasarca with periodical attacks of uremia, agurin somewhat increased the quantity of urine without augmenting the percentage of albumin. It had no effect, however, upon the uremic seizures, which finally subsided under the use of hot baths and diaphoretic measures. In the acute exacerbations of chronic nephritis the results were similar, while in chronic interstitial nephritis a considerable increase in the amount of urine voided was sometimes observed. From examinations of the urine during the administration of agurin I gained the impression that the solids were equally increased with the amount of fluid voided.

Briefly recapitulating my experience with agurin I would say that it is of special service in the dropsy of cardiac affections, sometimes useful in the ascites of cirrhosis and passive congestion of the liver, and also of occasional value for the removal of dropsical effusions left after the subsidence of acute inflammations of the kidneys, as well as in chronic nephritis without marked structural changes. In hydropericardium and hydrothorax the drug also serves a useful purpose in promoting the removal of fluid with or without paracentesis. As already mentioned the administration of agurin should be combined with the use of cardiac diuretics, such as digitalis or strophanthus, and diaphoretic measures, such as the hot bath and the vapor bath, with hydragogue cathartics.

707 West Wrightwood Avenue.

